# Wakakosha “You are Worth it”: reported impact of a community-based, peer-led HIV self-stigma intervention to improve self-worth and wellbeing among young people living with HIV in Zimbabwe

**DOI:** 10.3389/fpubh.2023.1235150

**Published:** 2023-07-28

**Authors:** Nadine Ferris France, Elaine Byrne, Owen Nyamwanza, Vongai Munatsi, Nicola Willis, Ronan Conroy, Sylivia Vumbunu, Moud Chinembiri, Samantha Maedziso, Munyaradzi A. Katsande, Takudzwa A. Dongo, Eimear Crehan, Webster Mavhu

**Affiliations:** ^1^Beyond Stigma, Dublin, Ireland; ^2^University College Cork School of Medicine, Department of Public Health & Epidemiology, Cork, Ireland; ^3^Centre for Positive Health Sciences, Royal College of Surgeons in Ireland, Dublin, Ireland; ^4^Centre for Sexual Health and HIV/AIDS Research (CeSHHAR), Harare, Zimbabwe; ^5^Renewal Trust, Harare, Zimbabwe; ^6^Zvandiri, Harare, Zimbabwe; ^7^Department of Epidemiology and Public Health, Royal College of Surgeons in Ireland, Dublin, Ireland; ^8^Community of the Work in Zimbabwe, Harare, Zimbabwe; ^9^The Work Under the Tree Trust, Harare, Zimbabwe; ^10^Speak Up, Sing Out, Kildare, Ireland; ^11^Department of International Public Health, Liverpool School of Tropical Medicine, Liverpool, United Kingdom

**Keywords:** HIV, inquiry-based stress reduction (IBSR), self-stigma, intervention, self-worth, wellbeing, young people, internalized stigma

## Abstract

**Introduction:**

Self-stigma—negative self-judgements or core beliefs—can result in feelings of shame, worthlessness and self-blame, and impacts social interaction, mental health and health service utilization among people living with HIV (PLHIV). Few interventions target self-stigma among PLHIV and, to our knowledge, none until now for adolescents and young people LHIV (AYPLHIV) in sub-Saharan Africa. We present qualitative findings on the perceived impact of a self-stigma intervention (*Wakakosha*, “You are Worth it”).

**Methods:**

The *Wakakosha* intervention adopted inquiry-based stress reduction (IBSR) at its core—a unique way of identifying and questioning deeply rooted self-stigma, combined with mindfulness, meditation and creativity. The intervention consisted of 16 × 3 hour group sessions. Supporting the intervention was a 156-page activity journal. We utilized a qualitative enquiry to explore the perceived impact of the intervention at various time points between November 2021 and November 2022, with 62 participants (*n* = 32 female). Discussions explored experiences of being involved in the intervention and any reports on changes in relation to self-stigma and shame. Additionally, we reviewed intervention documentation and creative elements. A thematic analysis guided generation of themes across all data sources.

**Results:**

Both intervention participants and coaches described the transformative effect of the intervention, detailing their experiences before and after. Main themes that emerged were positive changes around: self-confidence, self-agency, sense of purpose/meaning, body positivity, improved communication and personal/family relationships and, forgiveness. The intervention also transferred a set of practical skills on self-inquiry, mindfulness, meditation and creativity that continued to be used in participants’ daily lives.

**Conclusion:**

The *Wakakosha* intervention, using IBSR supported by music, creativity, writing and mindfulness techniques, showed potential for reducing self-stigma and improving self-worth among AYPLHIV. It also transferred practical skills to intervention participants and peer coaches, building their capacity to support others and deal with life challenges beyond HIV. The next phase is to continue supporting the young people to ensure fidelity as the peer coaches deliver the intervention to others. Study results indicate that culturally and practically, interventions to reduce self-stigma and/or improve self-worth operate at various levels and need to be designed and assessed at each level.

## Introduction

More than four decades into the global HIV pandemic, stigma remains a major barrier to HIV treatment, management and care (1–7). Goffman described stigma as, “an attribute that is significantly discrediting” which, in the view of others, serves to reduce the person who possesses it (8). Goffman also importantly identified stigma as a complex and multi-dimensional phenomenon (8). The internalization of stigma leads to what Goffman called “spoiled identity” or self-stigma (8, 9). HIV-related self-stigma is experiencing negative judgements towards oneself resulting in feelings of shame, worthlessness, self-blame and, emotional distress (10).

Self-stigma can result in non-adherence to HIV medication, lower quality of life and poor mental health (10, 11). A systematic review found that HIV-related stigma undermined antiretroviral therapy (ART) adherence by compromising general psychological processes (e.g., adaptive coping and social support), and 24/33 (73%) studies reported an association between HIV stigma and ART non-adherence (12). Multiple and intersecting drivers of stigma (HIV, orphaning, ill health, poverty) make adolescents and young people living with HIV (AYPLHIV) particularly vulnerable (13). Self-stigma contributes to AYPLHIV’s high rates of attrition from HIV treatment and care, treatment failure, morbidity and mortality (14–16). In sub-Saharan Africa, home to 85% of adolescents living with HIV globally, adolescents are the only population with increasing mortality rates (14, 17, 18).

While most stigma initiatives focus on averting discrimination (also known as enacted stigma) (9), the phenomenon of self-stigma is much less understood (11, 19). However, approaches that target self-stigma likely benefit psychological function and wellbeing of people living with HIV (PLHIV) in general and AYPLHIV, specifically (19). As many AYPLHIV were perinatally infected, they experience a great deal of self-stigma and shame throughout their young lives, including as a result of taboos about being sexually active (19). Of note, research suggests that self-stigma is experienced by PLHIV to a far greater extent than stigma received from the broader community (11, 20).

Self-stigma occurs at the individual level, but also influences interpersonal relationships, community participation and engagement and, ability to access rights and health services (11). We previously presented a framework comprising three interacting groups of factors (self, social, contextual) which influence the development and perpetuation of self-stigma (11). Further, the socio-ecological model provides a useful framework for understanding how health and wellbeing are influenced by multiple factors at various levels (individual, interpersonal, community, societal) (9, 21). However, few programs or interventions exist at the individual level and, to eliminate HIV stigma, it is necessary to work at multiple levels, including the individual, community and wider society (9, 22).

With reference to Zimbabwe, previous research has shown that AYPLHIV are particularly affected by stigma and self-stigma (13, 16, 23). Seventy-three per cent reported stigma and this affected their medication adherence; 47% had virological failure (16). In another study, AYPLHIV felt that because they were HIV positive, life was not worth living and described deliberately missing their medication so that they could eventually die (13). Further, in a study exploring the experience and manifestation of depression in AYPLHIV, adolescents attributed their experiences of depression to a sense of “being different from others”, both due to their HIV status and the impact HIV has had on their life circumstances (23). In the current study’s formative research with AYPLHIV (2021), all participants reported HIV-related self-stigma either occasionally or frequently and experiences of self-stigma reportedly led to poor wellbeing and decreased mental and physical health (19). At baseline, the Zvandiri-friendship bench trial of a community adolescent treatment supporters-delivered problem-solving therapy found 62% of adolescents LHIV at risk of common mental disorder. Intervention data confirmed recurring themes of low self-worth, shame and worthlessness (24).

Based on previous findings on the high prevalence and negative impact of self-stigma in AYPLHIV, coupled with the absence of individual-level interventions to address self-stigma (11, 22, 25), we developed an intervention to be layered within an existing program, Zvandiri. The Zvandiri (meaning “As I am”) program,^1^ recommended in 2013 by the World Health Organization as a best practice program (26), is a theoretically grounded, multicomponent, peer-led, differentiated service delivery model for children, AYPLHIV in Zimbabwe and adopted/adapted in 11 other countries (16, 27, 28). The program’s effectiveness in supporting improved HIV outcomes was recently demonstrated in the Zvandiri trial (adjusted prevalence ratio of virological failure or death 0.58, 95% CI 0.36–0.94; *p* = 0.03) (16). Zvandiri aims to directly improve AYPLHIV’s wellbeing and strengthen their engagement with services across the HIV treatment and care cascades, including through peer counselling, community outreach, digital health and support groups (27).

Evaluations have illustrated peer-support groups’ potential in significantly transforming AYPLHIV’s perception of HIV as a debilitating infection which they are experiencing in isolation, to a manageable condition (13, 28, 29). A key function of peer-support groups is that they nurture self-acceptance and enhance self-esteem through connectedness with others who have shared lived experiences (13, 28, 29). However, support groups do not intentionally address self-stigma and as a result, may not necessarily ameliorate it (19). Further, while young people do well in support groups, they continue to struggle outside the group setting (13, 29). A specific intervention addressing self-stigma is likely to additionally support AYPLHIV to deal with life challenges outside of peer-support groups. Informed by our formative study (19) and our previous work on self-stigma in Zimbabwe (10), we co-developed and piloted a culturally appropriate, locally informed intervention for AYPLHIV (18–24 years) to address self-stigma. Specifically, the intervention sought to increase self-worth and wellbeing by reducing self-stigma among AYPLHIV in Zimbabwe. This paper reports on findings from a qualitative study that explored the perceived impact of this intervention on self-stigma. For this pilot intervention, involving a relatively small sample, a qualitative exploration of nuanced and detailed accounts was most appropriate to provide a deeper understanding of any changes, how the intervention worked and what could be improved (see discussion).

## Materials and methods

### Study design: intervention overview

We sought to adapt and optimize an innovation we previously piloted among adults living with HIV in Zimbabwe—inquiry-based stress reduction (IBSR): the work of Byron Katie (10). IBSR is a unique way of identifying and questioning thoughts that cause shame, anger, fear and violence. Working with beliefs is imperative as self-stigma is deeply rooted in a person’s belief system. The beliefs that underlie shame, guilt, worthlessness and blame can be questioned with IBSR; allowing for new perspectives, insights and options that are kinder, more peaceful and empowering.^2^ IBSR provides an opportunity to discover a new and kinder way of living within oneself and the world—no matter what is happening. It uniquely combines in-depth inquiry of stressful thoughts with the process of cognitive diffusion and mindfulness. The evidence base for IBSR is growing and effectiveness has been shown on stressful beliefs in relation to cancer, HIV and among high school teachers (30–32).

The “Wakakosha: You are Worth it” project was a partnership between Zvandiri, Beyond Stigma and others. Local coaches, previously trained in IBSR, themselves adults LHIV, co-delivered the intervention together with an internationally IBSR-trained facilitator and an international creativity coach. In brief, the *Wakakosha* intervention aimed to equip AYPLHIV (aged 18–24 years) in the use of IBSR/self-inquiry to manage stressful beliefs, reduce self-stigma and grow their sense of self-worth and wellbeing. Following formative research in 2021 (19), a pilot 1 day workshop was held with 12 AYPLHIV and feedback systematically collected. The intervention was then fully designed based on the formative research, the pilot workshop and, drawing on previous self-stigma interventions designed for adults LHIV and survivors of gender-based violence (10). Principles underpinning the intervention included youth-centered, do no harm, flexible and adaptable, safeguard confidentiality, respect, non-discrimination, inclusive of key populations, playful and promoting best practice.

The *Wakakosha* intervention curriculum was designed (by NF and VM) to start with several universal stressful thoughts about being HIV positive and from there, to work systematically through more acute self-stigmatizing core beliefs at a deeper level each week. Introductory sessions on thinking, identifying and feeling emotions were also provided before progressing to the deeper issues in the curriculum. During the process, participants were encouraged to identify, and inquire about, their stressful thoughts regarding shame, HIV status, disclosure to others, sexuality, judgements of self/others, the body, illness and death. Overall, the intervention aimed to improve management of specific emotional and psychological symptoms (relating to self-worth, stress, anxiety, depression, fears). The intervention consisted of 16 × 3 hour group sessions which included individual and pair work (Table 1).

**Table 1:** Wakakosha session overview.

Session#	Session title
1	Self-stigma, emotions and me
2	The power of thinking
3	Introducing inquiry
4	Judgements about HIV
5	What others think of me
6	How HIV limits me
7	Deepening inquiry
8	ARVs and my story
9	My body
10	My body and sex
11	Forgiveness
12	Deepening inquiry
13	Shame and guilt
14	Relationships and fear of disclosure
15	Death and dying
16	My future

**Figure 1:**
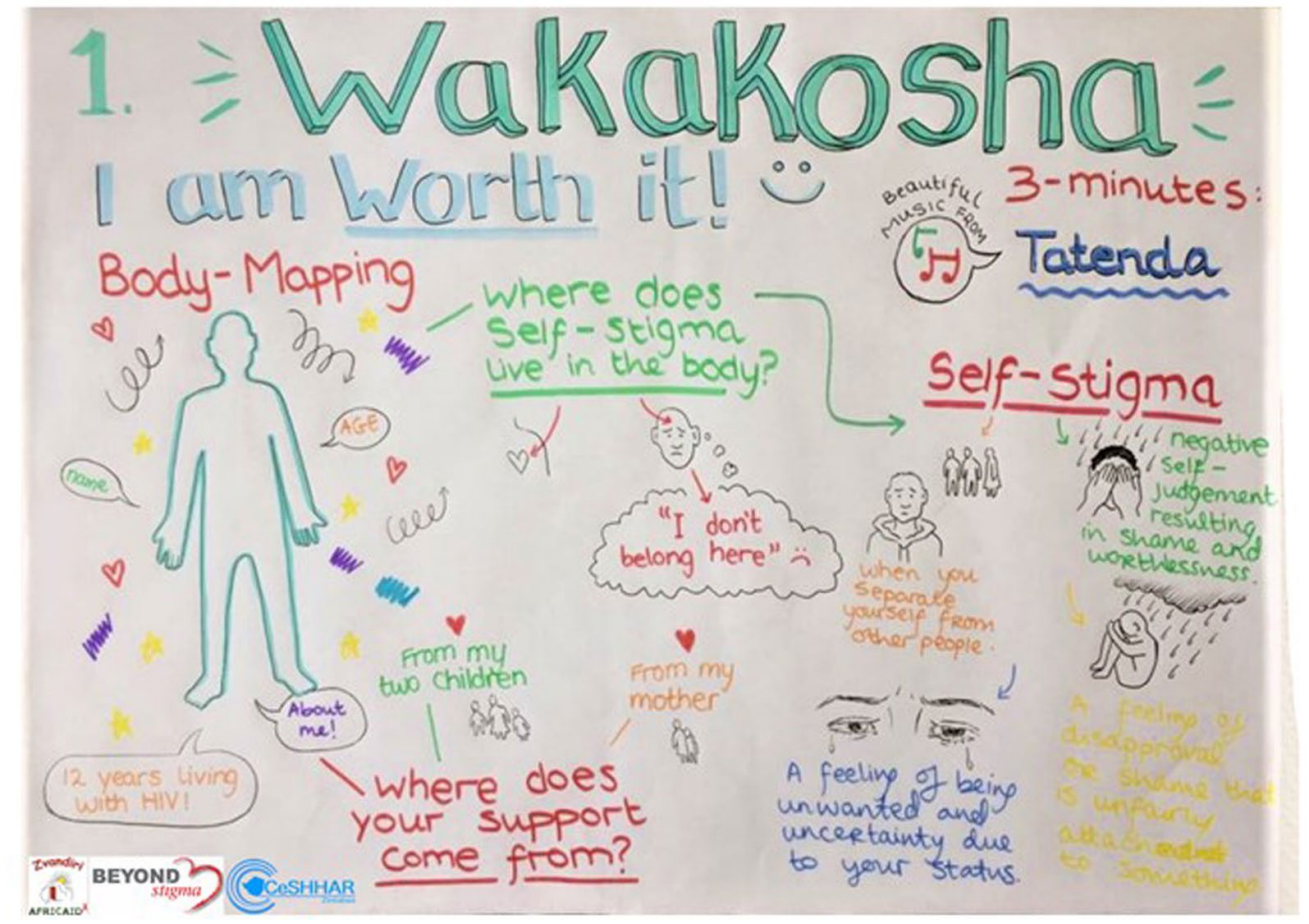
Wakakosha creativity example.

Each session contained a mix of theory, meditation, group and individual experiences of IBSR, music reflection and sharing of insights, with time for singing and songs carefully matched to a session’s themes to enhance learning and healing (see Figure 1). Supporting the intervention was a 156-page activity journal that contained worksheets, exercises and homework. Techniques such as journaling, box-breathing, podcasts, letter writing, poetry and drawing were used throughout. The intervention was first designed to be delivered as a 6 days residential face-to-face camp. Due to COVID-19, a hybrid of online and face-to-face was adapted and delivered over 16 weeks, with weekly homework and practice sessions supported by WhatsApp.

### *Wakakosha* intervention implementation

Thirty Zvandiri Community Adolescent Treatment Supporters (CATS) were initially recruited in 2021 to receive the intervention delivered by trained adult coaches (local and international). CATS are 18–24 years-olds LHIV, trained, mentored and supported to deliver structured support groups, counselling and tailored community-based adherence support to their peers (16, 28). From this group, 15 were identified to be subsequently trained as peer coaches, and attended a residential face-to-face, six-day immersive Training of Trainers in early 2022. Then a new group of 30 CATS was recruited and participated in the second delivery of *Wakakosha*, a 10 days face-to-face intervention (5 h per day), this time delivered mainly by peer coaches (supported by adult coaches). Each day of the training and/or intervention was evaluated, and feedback used real-time to adapt and ensure the curriculum was meeting the young people where they were. Towards the end, participants produced a song and recorded a music video which showed their experiences of the program and what happens when self-stigma is addressed. They also produced a podcast series through which they shared their own letters and poems about the body and forgiveness (available: https://www.beyondstigma.org/videos).

### Study design: qualitative evaluation

#### Study participants and research activities

A qualitative enquiry explored the perceived impact of the *Wakakosha* intervention at various time points. Firstly, in November 2021, a focus group discussion (FGD) was held with 12 of the 30 CATS who were the first to receive the intervention. Another 12 CATS took part in in-depth interviews (IDIs) between November 2021 and February 2022. Additionally, 12 of the 15 CATS recently trained as peer coaches took part in a FGD in March 2022. In addition, 24 of the 30 CATS recently trained by peer coaches took part in two separate FGDs in November 2022. We also conducted key informant interviews (KIIs) with 2 of the 4 adult coaches who had facilitated the intervention sessions (Table 2). Further, we analyzed intervention activities (e.g., letters to self or body, activity journals and daily evaluations).

**Table 2:** Summary of qualitative data collection.

Date	Participant category	Method	Number
November 2021	First set of CATS	FGDx1	12 (*n* = 6 female)
November 2021–February 2022	First set of CATS	IDIx12	12 (*n* = 6 female)
March 2022	Peer coaches	FGDx1	12 (*n* = 6 female)
November 2022	Second set of CATS (trained by peer coaches)	FGDx2	24 (*n* = 12 female)
November 2022	Adult local coaches	KIIx2	02 (*n* = 2 female)

#### Qualitative data collection and processing

Individual interviews and FGDs explored experiences of being involved, any changes in relation to self-stigma and shame (including examples of increased self-worth, examples of doing things they would not have done before, for instance: dressing better, enrolling for a course, approaching someone with confidence, self-agency, self-esteem, self-love) (see sample interview questions in Table 3).

**Table 3:** Sample interview questions for intervention participants by domain of interest.

Domain	Sample interview question
Overall experiences in program	Tell me about your experience in Wakakosha program. What did you like and dislike about the program?
Changes in thinking about self and HIV	What changed or stayed the same about how you think about yourself and HIV after taking part in this program?
Changes in self-stigma and shame	What changed or stayed the same about self-stigma and shame after taking part in this program?
Intervention components	Which components worked well or did not work well?
Intervention delivery	What do you think about the way the program was delivered? What worked well and what did not work?
Program recommendations	If the course were to be offered elsewhere, what should be maintained or changed? Why?

All discussions were led by two experienced Zimbabwean researchers (WM and ON) in private spaces and lasted approximately 30 min (individual interviews) and 1.5 h (FGD). All discussions were conducted in Shona, the participants’ language. With the participants’ permission, the discussions were audio-recorded. The recorded interviews were transcribed and translated verbatim into English. Interviewers (WM and ON) reviewed all transcripts to confirm their accuracy. A summary was written for each individual interview or FGD.

#### Qualitative data analysis and validation

Half of the interviews (*n* = 6) were thematically coded by two researchers (WM and ON), using deductive and inductive approaches by creating a coding framework based on the topic guides and adding codes iteratively (33). WM, ON, and EB met regularly to develop a common understanding of the codes and their application and, to discuss emerging patterns. These deliberations achieved *credibility* (ensuring researchers’ representation of themes fit with participants’ views) and *confirmability* (ensuring interpretations were clearly derived from the data) (34). Researchers used memoing to consolidate emerging themes and as a reflexivity tool, whereby they kept a “self-critical” account of the research process and reflected on potential biases to enhance findings’ *dependability* (34).

Any additional codes identified from this first set of transcripts were added to the coding framework. Transcripts were entered into NVivo 14 (QSR International, Melbourne, Australia) and fully coded using the modified coding framework; care was taken to identify any additional emerging codes. Codes were grouped and emerging themes were identified and supported with verbatim quotes. A workshop was held with three peer coaches (SM, MK, and TD) and two adult coaches (SV and MC) to review research findings and contribute to manuscript writing.

## Results

Between November 2021 and November 2022, sixty young people LHIV, aged 18–24 years took part in the research. Of these, 30 (50%) were female. We also held discussions with two of the four female adult coaches (see Table 2).

Overall, both intervention participants and coaches described the transformative effect of the *Wakakosha* intervention, detailing their experiences before and after. The main themes that emerged were the existence of previous self-stigmatization and the move to positive changes around: self-confidence and self-agency, sense of purpose and meaning in life, body positivity, improved communication and personal/family relationships, self-forgiveness and forgiveness of others. These themes are described in detail below.

### Transitioning from previous self-stigmatization

Participants described how they experienced self-stigma, including negative self-judgements, prior to enrolling onto *Wakakosha*. One participant noted that there were “…*certain things that might happen. Simply because you are HIV positive you will begin to personalize issues*… ‘*So this person did not see me when I was passing by*…*Has she heard something about me*?’ *You might be assuming that someone has ignored you, yet this is all in your mind*…” (Male 23 years, FGD). Participants also described instances of negative self-judgement. “…*Telling yourself that you cannot do this because of your* (*HIV*) *status*” (Female 23 years, FGD).

Participants described effects of these self-stigmatization experiences including self-isolation and depression. “*When I was home and we had visitors, I would shut myself in a room because I am HIV positive*…*I was worried that visitors would see me and guess my status. I was worried that family members would disclose my status to others. So, you are always alone in your room, and this causes so much stress because you are always assuming several issues*” (Female 20 years, FGD). One participant described how similar distressing experiences triggered perpetual suicidal thoughts. “*I was only thinking about death…It was better for me to die as there was no reason for me to continue living. My relationships were difficult and there were also challenges at home because of my* (*HIV*) *status*” (Female 19 years, IDI). Prior to the intervention, participants not only *agreed* with stereotypes or negative perceptions about themselves but *applied* them too, including the belief that they could not amount to anything.

### Experiences after the intervention

#### Self-confidence and self-agency

Intervention participants described gaining various skills including self-confidence. This often prompted them to pursue previously “impossible” goals such as enrolling for academic and professional courses. “*I encouraged her, and she enrolled for a teacher training course*” (adult coach, KII). Another participant reported pursuing a career as a nurse aide. Some participants reported renewed self-agency. A participant who had inherited a piece of land but was not using it productively due to self-stigma-induced loss of self-agency reclaimed it and started farming, remarking that, “*I would tell myself that there was no way I could farm that whole piece of land…If I had not attended Wakakosha I would have surrendered the land to someone else*” (Female 24 years, FGD).

#### Sense of purpose and meaning in life

After the intervention, participants affirmed a new sense of purpose in their lives as reflected by one who noted that, “*After the training I learnt that there is nothing I cannot do, and I can do it*” (Male 21 years, FGD). More so, the intervention inspired a sense of self-worth and purpose in the participants, with some rediscovering their entrepreneurial passions. Another participant labelled themselves “a jack of all trades” attesting to a renewed entrepreneurial drive and passion for new vocations, stating that, “*I am now involved in five things…welding, plumbing, art, building and fashion*” (Male 23 years, FGD).

#### Body positivity

Because of self-stigma, participants, especially female ones, developed negative attitudes towards their bodies as evidenced by reports of having previously been ashamed of, and neglecting, their own bodies. A participant remarked that, *I used to hate my body, and I would say,* “*I do not want my body because I am tiny. I do not want my skin because it is too dark*” (Female 20 years, FGD). Yet another participant narrated, “*I would ask myself why I was not gaining weight…my body was too thin. When I looked at other girls with bigger body structures, I would ask myself why I could not also get there*” (Female 23 years, FGD). Participants further acknowledged different ways in which they mistreated their bodies, with one admitting to their body that they, “*hurt you* (*body*) *terribly by not caring well and starving you because of my self-stigma…*” (Dear Me Podcast #4), while others engaged in alcohol and drug abuse as well as deliberate self-poisoning in a bid to kill themselves (Dear Me Podcast #8, #13).

Such thoughts, however, changed upon completing *Wakakosha*. Noteworthy was the body positivity message that the intervention inculcated in participants, motivating them to reevaluate their beliefs and attitudes towards their bodies and embracing body positive routines. One participant remarked that, “*It was only after Wakakosha when I realized that I should be proud of my body because my face has not been reacting to the drugs that I am taking*” (Female 23 years, IDI). More so, a recurring message in the Dear Me Podcasts is that of participants committing to taking better care of their bodies through eating healthy, quitting alcohol and drug abuse as well as improved hygiene (see Dear Me Podcast #11, #12, #13).

#### Improved communication and personal/family relationships

Participants also reported improved communication and personal relationships. As participants were relatively young (18–24 years), forming relationships was one of their key considerations. Nearly all described previously struggling to come to terms with their emerging sexuality in the context of a life threatening, sexually transmissible infection. *I would tell myself that* “*I am HIV positive; I cannot date someone who is HIV negative*” (Female 22 years, FGD). Even when they were now in relationships, participants would worry about family size and “curtailed” longevity. “*I was stressed. When I gave birth, my sister-in-law said* ‘*You should not have another child because you are going to die soon. Who will take care of your children?*’ *So, I had told myself that it is fine to just have one child. I held on to those words*…” (Female 21 years, FGD).

Post intervention, participants reported improved personal and family relationships. Most participants reported previously being bitter and angry towards their parents, relatives, and other members of the community. One participant reported that after the intervention relations thawed between him and his father, admitting that “…*now* (*after the intervention*) *I have realized that I am now more humane, I am no longer exchanging* (*harsh*) *words and I now respect him*…” Another participant was elated with how his love life had improved, stating that, “*My love life is now great. Yesterday was a wow for me. We were coming from church, and she invited me for dinner. It was excellent. My love life is now great*” (Male 22 years, FGD).

#### Self-forgiveness and forgiveness of others

Many participants reported positive changes in self-forgiveness and in forgiveness of parents for passing on HIV. Most were bitter towards their parents but through the intervention, they were able to forgive and erase the negative thoughts, “*Even to my dad, I used to believe he was stigmatizing me when I was young…he did not visit me. So, I just wrote a letter to both asking for forgiveness. I learnt about forgiveness because I was very bitter towards my parents about being the only child who was born with HIV in my family*” (*Male 18* years*, IDI*). Through the intervention’s transformative effect, participants also learnt self-forgiveness as evidenced in the powerful messages of forgiveness in the Dear Me Podcast series. Participants also asked for forgiveness for having put their bodies on the line by starving themselves and abusing alcohol (Dear Me Podcast #5, #13) while others forgave themselves for ignorantly indulging in risky sexual escapades (Dear Me Podcast #6, #11).

A male participant described how the *Wakakosha* intervention had helped him find closure. “*So, I wrote a letter* (*as part of intervention activities*) *to both of them* (*deceased parents*) *asking for forgiveness*…” (Male 22 years, IDI). He went on to describe what he did next. “*One weekend I travelled to the rural areas, bought flowers, and went to their graves and* ‘*said*’ *to them* ‘*I am very sorry for not forgiving you all along…*’ *I then placed the flowers on their grave*” (Male 22 years, IDI). Another male participant stated, “*I wrote a letter to my mother asking for forgiveness. I was angry due to my status, saying* ‘*I am HIV positive because you were careless. You were careless with your life, and you gave birth to me fully aware of your status.*’ *So, I wrote the letter asking for forgiveness because I was ignorant*” (Male 19 years, IDI).

Some letters were addressed to surviving relatives. “*…She was blaming her uncle* (*father*’*s young brother*) *for ill-treating her after her father*’*s death. But after doing the session, she realized that this uncle did all he could to provide for her and her siblings. For example, he paid their school fees. She wrote an* “*amendment letter.*” *She was now saying that she has actually realized that her uncle was not a bad person. He was actually a good person for taking care of her. As an uncle, he was not obliged to look after her when her parents passed on. So she realized that she had to thank him*” (peer coach, IDI).

#### Experiences about the approach/process

Both intervention participants and coaches (facilitators) described an overwhelming transformative effect of the intervention. CATS described for example, how they often spend time and effort offering peers support around adherence and overall wellbeing support, and rarely get the opportunity to introspect. Despite their mentorship, training, and support, they reported feeling unable to address personal challenges including self-stigma. One CATS summed up this shortcoming through a local proverb “*N*’*anga haizvirapi*” (*A traditional healer cannot treat themselves*) (Female 24 years, IDI), an expression which underscores that CATS need support too. “*Also, it helps us the CATS. Yes, we might be trained but, in some instances, you will be focused on other people. But with Wakakosha you are also focusing on yourself*” (Male 24 years, IDI).

Participants described in detail the new skills they had acquired. “*We were taught that if there are challenges, we should not see these as permanent. We should have positive and not negative thoughts only. For example, when faced with a situation, we could only view it from the negative side, without looking at the positive side. Through this program* (*Wakakosha*) *I was taught to look at both sides and then think about how I can overcome the problems that I am facing*” (Male 23 years, IDI). As with other participants, he found this problem-solving skill quite empowering.

CATS were also equipped with vital skills they now use to help their peers. *I have skills that I am using on other people. There is one called box breathing. We would do this box breath whenever we were starting sessions. You would close your eyes and breath in and out… breath in and out. I normally use this when I am with a client who is disturbed and is facing some challenges. Maybe he is not calm…I can also ask them to give me their emotions from 1–10. By so doing, you will realize that I am feeling this emotion because of this and that. We will then start the counselling session* (Male 24 years, IDI).

Commenting on the activity journal, a participant stated “*…In the* ‘*journal,*’ *there were some* ‘exercises,’ *and these were part of homework. You would ask yourself through the sheets the emotions that you were going through on that particular day. You would then circle. From there, you would then start doing an inquiry using that yellow card that contained the four questions and the turnaround prompts. And then stressful thoughts that caused you to be in that emotional state will begin to emerge. And then at the end of the day you will be in a different emotion*” (Male 22 years, IDI).

The acquired skill sets were not only helpful to intervention participants but also others. “*So, the skills that I am using on other people include inquiry and the yellow card. Out there, there are peers who might be having stressful thoughts. Sometimes when I contact a client, I will end up using that yellow card*” (Male 23 years, IDI). Another participant highlighted how components of the intervention can be used beyond self-stigma situations, noting that, “…*When I go home and I start doing this with my aunt* (*55 years old*)*, asking her to turn around her thoughts. Holding the yellow card and asking her about her thoughts on that issue. I will then ask her to do the turnaround thoughts… turn around to yourself. At the end of the day, she will start laughing asking if these were the activities we were doing, and I would say* ‘*Yes*’” (Male 22 years, IDI). Overall, participants felt the skills they learned could be applied beyond self-stigma into their everyday lives, in particular the ability to solve problems.

Lastly, participants reported how creativity (music, drawing, poetry) helped them engage in challenging topics on self-stigma, thus promoting awareness and healing. “*The singing helped us easily share our feelings whether sad, happy or angry*” (Female 18 years, FGD) (creativity component of this intervention to be reported separately).

#### Delivery challenges and recommendations

As already noted during the description of intervention delivery, due to COVID-19, some sessions had to be delivered online, which had its own challenges. Participants articulated some of the challenges. “*…Going forward, we will prefer face to face mentoring. There are instances when things are said, and you have to express your emotions…If it is online, no one can see what is going on. If you are discussing face to face, you can express your emotions and then you receive counselling*” (Male 21 years, FGD). A local adult coach weighed in, “*Especially when we were still doing this from home* (*online*)*, it was worse. It was okay when we were coming to Zvandiri because they have a strong network connection. But from home, you could see the young people complaining that there is no power and this affects network connectivity. Therefore, I did not like the online program at all, especially during the first days when everything was to be done online*” (adult coach, KII). Implementing an online intervention within a context characterized by erratic power supply, limited internet connectivity and exorbitant data charges involves “scaling a mountain.”

Some recommendations centered around ensuring intervention materials taken home do not result in inadvertent HIV status disclosure. “*I would like to go back to* (*activity*) *journals. Those are for us participants who are attending the meetings. However, there is over emphasis on HIV. Yes, it*’*s good but I might be staying at a home where relatives and friends come. Maybe I have not yet disclosed to them. Through this CATS program, we have disclosed, and we are now used to this. But some people might be affected when they are going around with that* ‘*book*, *and someone opens the* ‘*book,*’ *they will see text which reads,* ‘*Because of HIV….*’ *If only they could find other words to talk about HIV in a way other people will not know HIV is being discussed*” (Male 24 years, FGD). These concerns should be taken into consideration going forward.

## Discussion

We qualitatively explored the perceived impact of “Wakakosha: You are Worth it,” a culturally appropriate, locally informed intervention for AYPLHIV to address self-stigma. The evaluation revealed that the *Wakakosha* intervention effected positive changes on many aspects of self-stigma including self-judgements, self-worth, sense of purpose, self-agency in career and education, body image, self-forgiveness and forgiveness of others and improved communication with family and significant others. It also transferred a set of practical skills on self-inquiry, mindfulness, meditation and creativity that continued to be used in participants’ daily lives. We previously noted how interventions to support PLHIV in dealing with self-stigma, particularly at the level of core beliefs, are lacking (11, 22, 25). Interventions such as *Wakakosha*, tailored to the context of HIV self-stigma and using knowledge of underlying core beliefs, may better equip participants with the psychosocial tools and strategies to mitigate self-stigma.

The emerging field of positive health perceives health as a state of wellbeing, not an illness and can be defined as an approach to health that recognizes factors that drive disease and illness and focuses on the processes that underpin good health and wellbeing (35–39). From our study findings, we can see many aspects of positive health emerging and being strengthened (e.g., gaining self-confidence, a new sense of purpose in life, a sense of self-worth, self-agency and purpose). These and other findings (39, 40) buttress calls for positive mental health interventions for AYPLHIV that are anchored upon the need to improve and evaluate human strengths and capabilities as enshrined in positive outcomes such as self-esteem, self-worth and flourishing (35–37).

In addition, what emerged from our study were also a number of cultural and contextual insights into positive health, namely the culture of discussing feelings and emotions, spirituality, and individual versus collective agency. In relation to feelings and emotions, in Zimbabwe, as in other sub-Saharan settings, culturally speaking or articulating feelings and emotions is uncommon (28). Importantly, the intervention reportedly had a positive psychosocial effect on males who in this context are less able to discuss or express emotional concerns, largely due to hegemonic masculinity where males are socialized around toughness and concealing emotions (41, 42). Cultural sensitivity in ways of doing this needed to be incorporated into the training.

What also emerges is the strong collectivist culture in Zimbabwe and thus the wellbeing of others is as, if not more, important for positive health. In positive psychology, as noted by Wong (43), there has been some inclusion of altruism (44) and compassion (45), but “there are cultural differences in the balancing act between *me* and *we*. … we need to emphasize positive motivations, processes, activities, and outcomes for both individuals and groups” (Wong, 2011, p. 72). For an individual in Zimbabwe, as in most of sub-Saharan Africa, self-acceptance is bound up with acceptance within one’s community. Hence, for the AYPLHIV, it was difficult for them to separate themselves from the diminished status of their parents due to HIV infection, increasing the potency of the shame and self-stigmatizing beliefs they had about themselves and their parents. These difficulties of separating the self from others also strengthens the value of community and opinions of others regarding morality and HIV acquisition, which in turn fuels shame, self-stigma and the acceptance of social stigma and discrimination as well-deserved punishment for sexual deviance (13, 19). Therefore, addressing self-stigma at individual and communal levels helps to defuse disempowering narratives in the individual first and this cascades to the community.

Participants reported changes at the household/family level as a result of the intervention. A potential limitation of this study was that we did not directly collect data at this level. In keeping with the socio-ecological model (21), as per the information reported by respondents, it is likely that a successful intervention at the individual level will have a positive impact at household/family level. Future explorations should explore changes beyond the individual more systematically.

Increasing attention continues to be paid to community-based peer-support programs as a mechanism to improve clinical outcomes and wellbeing (28). While the predominate focus of research on peer support for AYPLHIV has focused on the potential benefits to those receiving peer support (26), there is also growing recognition of the essential need to focus on the health and wellbeing of the peer supporters themselves (46). This study demonstrates that peer supporters themselves also benefit from the various intervention activities. Ensuring support and ongoing mentoring for the peer coaches is key to the ongoing success of this and other interventions (28). As part of *Wakakosha*, an online Toolkit has been produced and will be tested to provide this support to the peer coaches going forward.

Creativity clearly played a supportive role in the absorption, emotional processing and deepening of positive changes around self-stigma and shame, variables which are difficult to openly discuss without causing or triggering deep hurt or stressful thoughts around experiences within oneself and with others. Our team has previously used creativity on other difficult topics, including to explore experience and manifestation of depression in ALHIV (23). Further exploration is needed to specifically measure and assess the impact of creativity on self-stigma and shame among AYPLHIV.

Our assessment was intentionally qualitative as though we could have used some quantifiable measures of program impact, these have a number of drawbacks. Firstly, they limit the number of domains that can be assessed; most of the outcomes as described above would not have been captured by standard measurement scales. Secondly, it is not possible with measurement scales to interpret differences in mean scores in terms of the lived experience of the participants. For example, a change in mean score over a group of participants does not tell us what proportion of participants materially benefitted. Comparing scales across different cultures has been likened to comparing chopsticks with forks if appropriate adjustments in scales are not incorporated (47). Lastly, the qualitative approach respects the agency of the participants. Participants entered the program with their own individual hopes and aspirations. These were captured in an early activity called “This is Me” embedded in the curriculum. We took the decision to assess the program relative to these individually valued outcomes rather than against universal ones.

Another limitation is that, as participants were already Zvandiri CATS with potentially lower self-stigma levels, the overall positive impact found here may not necessarily reflect what would occur in for example, CATS clients. Future initiatives should evaluate impact of the intervention when it is offered to CATS’ clients and AYPLHIV outside of the Zvandiri program. In addition, despite confidentiality assurances, participants may have felt obligated to largely report positive experiences whilst downplaying negative ones.

## Conclusion

This intervention shows potential for reducing self-stigma and improving self-worth among AYPLHIV. The next phase of this intervention will be to continue training and supporting the young people to ensure fidelity and provide quality support as the peer coaches deliver the intervention to others. Ongoing support and mentoring for the peer coaches will take time and resources, particularly in the first few years of gaining traction for a new methodology such as IBSR. As this is a cognitive and mindfulness-based methodology, careful supervision and ongoing individual practice is important. Future research and evaluation should carefully consider spirituality and the communal view of positive health more broadly when looking at self-stigma and self-worth. It should also be cognizant of the cultural context and consider using the socio-ecological framework to reflect the various levels affected - the individual, family and wider society.

## Data availability statement

The original contributions presented in the study are included in the article/supplementary material, further inquiries can be directed to the corresponding author.

## Ethics statement

The studies involving human participants were reviewed and approved by Medical Research Council of Zimbabwe (#2604). The patients/participants provided their written informed consent to participate in this study.

## Author contributions

All authors listed have made a substantial, direct, and intellectual contribution to the work and approved it for publication.

## Conflict of interest

The authors declare that the research was conducted in the absence of any commercial or financial relationships that could be construed as a potential conflict of interest.

## Publisher’s note

All claims expressed in this article are solely those of the authors and do not necessarily represent those of their affiliated organizations, or those of the publisher, the editors and the reviewers. Any product that may be evaluated in this article, or claim that may be made by its manufacturer, is not guaranteed or endorsed by the publisher.

## Footnotes

^1^ www.zvandiri.org

^2^See www.beyondstigma.org for description of IBSR methodology.
